# Metallothionein synthesis increased by Ninjin-yoei-to, a Kampo medicine protects neuronal death and memory loss after exposure to amyloid β_1-42_

**DOI:** 10.1186/s40780-022-00257-8

**Published:** 2022-11-01

**Authors:** Haruna Tamano, Haruna Tokoro, Daichi Murakami, Rin Tsujimoto, Yuka Nishijima, Erina Tsuda, Satoshi Watanabe, Miki Suzuki, Atsushi Takeda

**Affiliations:** School of Pharmaceutical Sciences, University of Shizuoka, 52-1 Yada, Suruga-ku, Shizuoka, 422-8526 Japan

**Keywords:** Metallothionein, Amyloid β_1-42_, Alzheimer’s disease, Zn^2+^ dysregulation, Ninjin-yoei-to, Kampo medicine

## Abstract

**Background:**

It is possible that increased synthesis of metallothioneins (MTs), Zn^2+^-binding proteins is linked with the protective effect of Ninjin-yoei-to (NYT) on Zn^2+^ toxicity ferried by amyloid β_1-42_ (Aβ_1-42_).

**Methods:**

Judging from the biological half-life (18-20 h) of MTs, the effective period of newly synthesized MT on capturing Zn^2+^ is estimated to be approximately 2 days. In the present paper, a diet containing 3% NYT was administered to mice for 2 days and then Aβ_1-42_ was injected into the lateral ventricle of mice.

**Results:**

MT level in the dentate granule cell layer was elevated 2 days after administration of NYT diet, while the administration reduced intracellular Zn^2+^ level increased 1 h after Aβ_1-42_ injection, resulting in rescuing neuronal death in the dentate granule cell layer, which was observed 14 days after Aβ_1-42_ injection. Furthermore, Pre-administration of NYT diet rescued object recognition memory loss via affected perforant pathway long-term potentiation after local injection of Aβ_1-42_ into the dentate granule cell layer of rats.

**Conclusion:**

The present study indicates that pre-administration of NYT diet for 2 days increases synthesis of MTs, which reduces intracellular Zn^2+^ toxicity ferried by extracellular Aβ_1-42_, resulting in protecting neuronal death in the dentate gyrus and memory loss after exposure to Aβ_1-42_.

## Background

In the Alzheimer’s disease (AD) pathogenesis, neuronal accumulation of amyloid β_1-42_ (Aβ_1-42_), a causative peptide causes synaptic and neuronal losses, which affect hippocampus-dependent memory [1, 2]. In persons with mild cognitive impairment prior to the AD pathogenesis, approximately 30% neurons are lost in the entorhinal cortex and induce synaptic loss to the dentate gyrus. The loss is correlated with cognitive impairment [1, 3] and the perforant pathway-dentate granule cell synapse is an earliest site affected in Aβ_1-42_-mediated pathogenesis [4]. Aβ_1-42_ readily captures Zn^2+^ in the extracellular fluid and Zn-Aβ_1-42_ complexes are preferentially taken up into dentate gyrus neurons, resulting in cognitive impairment and neuronal death, which are linked with intracellular Zn^2+^ toxicity ferried by Aβ_1-42_ [5–7]. The protection of dentate gyrus neurons against Zn^2+^ toxicity is a potential target to protect the Aβ_1-42_-mediated pathogenesis [8, 9].

Cholinergic degeneration in the brain is associated with AD pathophysiology and maintenance of choline acetyltransferase activity is benefit to the patients with AD [10–12]. It has been reported that donepezil a cholinesterase inhibitor, is effective for the symptom alleviation of the patients with AD [13, 14]. Furthermore, the treatment with both donepezil and Ninjin-yoei-to (NYT), a Kampo medicine more than 2 years ameliorates cognitive performance and alleviates AD-associated depression [15]. However, there is no report that NYT itself is effective on the AD pathophysiology. We have reported that neuronal death in the dentate gyrus induced by Aβ_1-42_ is protected by pre-administration of NYT for 14 days [16]. In the present study, we presumed that NYT-induced synthesis of metallothioneins (MTs), Zn^2+^-binding proteins, which may reduce intracellular Zn^2+^ toxicity by Aβ_1-42_, contributes to the protective effect. On the basis of the data on the biological half-life (18–20 h) of MTs [17], we orally administered NYT diet to mice for 2 days and tested the protective effect on neuronal death in the dentate gyrus. Because intracellular Zn^2+^ toxicity by Aβ_1-42_ in the dentate gyrus also affects object recognition memory [5], we also checked the effect of NYT diet on memory loss.

## Material and methods

### NYT diet

NYT obtained from Tsumura & Co. (Tokyo, Japan) was in the form of dried powder extract. NYT was prepared from a mixture of Angelicae radix (4.0 g, root of *Angelica acutiloba* Kitagawa), Hoelen (4.0 g, fungus of *Poria cocos* Wolf), Rehmanniae radix (4.0 g, root of *Rehmannia glutinosa* Lib., var. purpurea Mak), Atractylodis rhizoma (4.0 g, root of *Atractylodes japonica* Koidzumi), Ginseng radix (3.0 g, root of *Panax ginseng* C.A.Mey), Cinnamomi cortex (2.5 g, bark of *Cinnamomum cassia* Bl.), Aurantii nobilis pericarpium (2.0 g, peel of *Citrus unshiu* Markovich), Polygalae radix (2.0 g, root of *Polygala tenuifolia* Willd), Paeoniae radix (2.0 g, root of *Paeonia lactiflora* Pall), Astragali radix (1.5 g, root of *Astragalus membranaceus* Bge.), Glycyrrhizae radix (1.0 g, root of *Glycyrrhiza uralensis* Fisher) and Schisandrae fructus (1.0 g, fruit of *Schisandra chinensis* Baill). A diet containing 3% NYT was prepared by Oriental Yeast Co. Ltd. (Yokohama, Japan). A control diet without NYT was also administered to mice and rats in place of NYT diet. The direct administration via mouth is better for a more accurate dosage, while it was difficult to prepare such an aqueous solution of NYT for administration because of the solubility. The administration as a NYT diet was selected in the present study.

### Animals

Male ddY mice (10 weeks of age) and Male Wistar rats (10 weeks of age), which were obtained from Japan SLC (Hamamatsu, Japan), freely access a control diet, a 3% NYT-containing diet, and water. NYT diet did not modify the body weight of mice 4 weeks after administration because of the almost the same intake between the control and NYT diets in the previous study [16]. Body weight of mice and rats was also almost the same between intakes of the control and NYT diets in the present experiments. All the experiments were performed in accordance with the Guidelines for the Care and Use of Laboratory Animals of the University of Shizuoka. The Ethics Committee for Experimental Animals has approved the present study in the University of Shizuoka.

### Intracerebroventricular (ICV) injection of Aβ

Saline (vehicle) and Aβ_1-42_ (ChinaPeptides, Shanghai, China) in saline (25 µM) was delivered into the lateral ventricle of mice at the rate of 0.5 µl/min for 40 min (500 pmol/mouse) via a microinjection canula as described previously [16].

### MT immunostaining

**Fig. 1:**
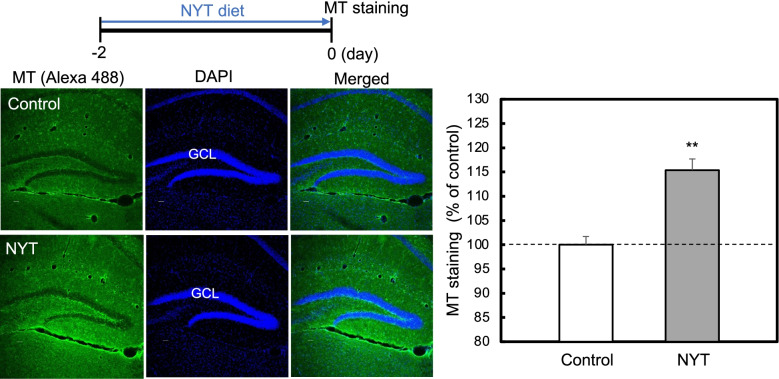
MT level is elevated 2 days after administration of NYT diet. MT immunostaining was determined in the dentate granule cell layer (GCL) 2 days after administration of NYT diet to mice (left). The data (mean ± SEM) indicate the rate (%) of MT staining after NYT administration to that after the control diet administration that was indicated as 100% (right). **, *p* < 0.01, vs. control (*t*-test). Control, 16 slices from 4 mice; NYT, 20 slices from 5 mice

**Fig. 2:**
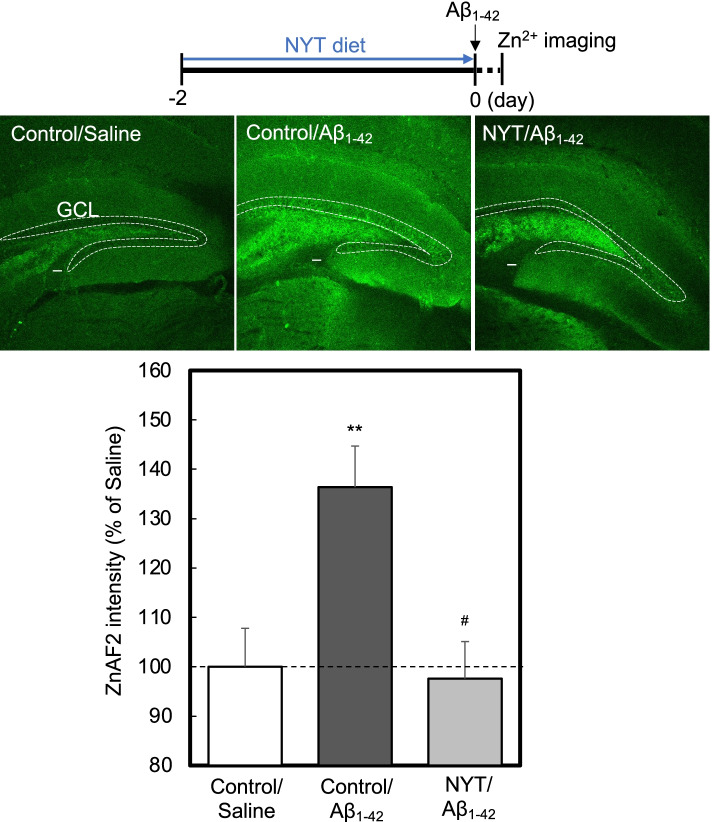
Administration of NYT diet cancels intracellular Zn^2+^ level increased by Aβ_1-42_. Intracellular ZnAF-2 fluorescence was determined in the granule cell layer (GCL) of mice 1 h after ICV injection of Aβ_1-42_ (upper). Bar; 50 µm. The data (mean ± SEM) indicate the rate (%) of ZnAF-2 fluorescence after Aβ_1-42_ injection to that after saline (vehicle) injection that was indicated as 100% (lower). **, *p* < 0.01, vs. saline: ^#^, *p* < 0.05, vs. Aβ (Tukey’s test). control/saline, 32 slices from 8 mice; control/Aβ, 29 slices from 8 mice; NYT/Aβ, 16 slices from 4 mice. Bar; 50 µm

**Fig. 3:**
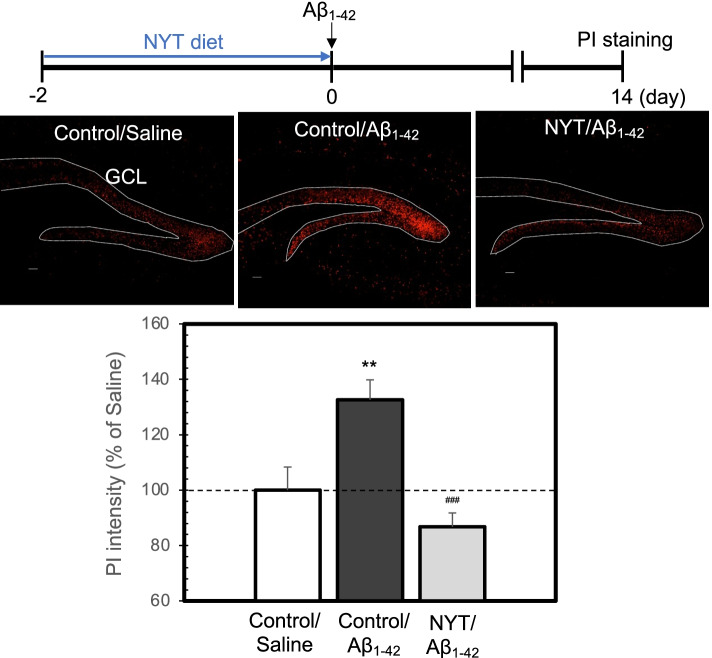
Neuronal death assessed by PI staining is rescued by NYT diet. PI fluorescence was measured in the granule cell layer (GCL) of mice surrounded by the dotted line 14 days after ICV injection of Aβ_1-42_ (upper). Bar; 50 µm. The data (mean ± SEM) indicate the rate (%) of PI fluorescence after Aβ_1-42_ injection to that after saline (vehicle) injection that was indicated as 100% (lower). **, *p* < 0.01, vs. control/saline, ^###^, *p* < 0.001, vs. control/Aβ (Tukey’s test). control/saline, 8 slices from 3 mice; control/Aβ, 8 slices from 3 mice; NYT/Aβ, 14 slices from 5 mice

**Fig. 4:**
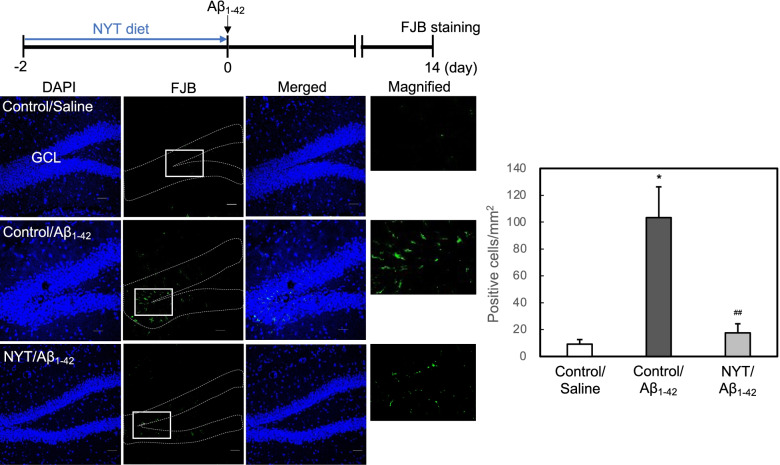
Neuronal death assessed by FJB staining is rescued by NYT diet. Brain slices were stained with FJB and DAPI. FJB fluorescence was measured in the granule cell layer (GCL) of mice surrounded by the dotted line 14 days after ICV injection of Aβ_1-42_ (left). Magnified images are the areas from the white square in FJB images. Bar; 50 µm. FJB positive cell number was counted in the GCL. The data (mean ± SEM) indicate FJB-positive cells in the unit area after injection of vehicle or Aβ_1-42_ (right). *, *p* < 0.05, vs. control/saline, ^##^, *p* < 0.01, vs. control/Aβ (Tukey’s test). control/saline, 12 slices from 4 mice; control/Aβ, 36 slices from 12 mice; NYT/Aβ, 10 slices from 4 mice

A 3% NYT-containing diet was administered to mice for 2 days. The mice were anesthetized with chloral hydrate and perfused with ice-cold 4% paraformaldehyde in PBS, followed by removal of the brain and overnight fixation in 4% paraformaldehyde in PBS at 4 °C. Fixed brains were cryopreserved in 30% sucrose in PBS for 2 day and frozen in Tissue-Tek Optimal Cutting Temperature embedding medium. Coronal brain slices (30 µm) were prepared at -20 °C in a cryostat, picked up on slides, adhered at 50 °C for 60 min, and stored at -20 °C. For immunostaining, the slices were first immersed in PBS for washing, incubated in blocking solution (3% BSA, 0.1% Triton X-100 in PBS) for 1 h, and rinsed with PBS for 5 min followed by overnight incubation with anti-MT antibody [UC1MT] ab12228 (Abcam) in 0.1% Triton X-100 in PBS (1:200 dilution) at 4 °C. The slides were rinsed with PBS for 5 min three times and incubated in blocking buffer containing Alexa Fluor 488 goat anti-mouse secondary antibody (Thermo Fisher Scientific) in 3% BSA, 0.1% Triton X-100 in PBS (1:200 dilution) for 3 h at room temperature. Following three rinses in PBS for 5 min, the slides were bathed in 0.1% DAPI in PBS for 5 min, rinsed with PBS for 5 min three times, mounted with Prolong Gold antifade reagent, and placed at 4 °C for 24 h. Immunostaining images were measured in the dentate gyrus using a confocal laser-scanning microscopic system (Ex/Em: 495 nm/519 nm) (Nikon A1 confocal microscopes, Nikon Corp.) as described previously [16]. To obtain the best fluorescence images and measure the difference in fluorescence intensity among groups exactly, we first checked the relationship between the gain (fluorescence sensitivity) and fluorescence intensity and then carefully decided the best gain for measuring the exact changes in fluorescence intensity. This decision was separately performed in all experiments (Figs. 1, 2, 3 and 4).

### In vitro ZnAF-2 imaging

Aβ_1-42_ (25 μM) in saline was intracerebroventricularly injected via a microinjection canula at the rate of 0.5 µL/min for 40 min (500 pmol/mouse) of anesthetized mice. One hour after the start of injection, coronal brain slices (400 µm) were prepared in ice-cold choline-Ringer solution containing 124 mM choline chloride, 2.5 mM KCl, 2.5 mM MgCl_2_, 1.25 mM NaH_2_PO_4_, 0.5 mM CaCl_2_, 26 mM NaHCO_3_, and 10 mM glucose (pH 7.3) to suppress excessive neuronal excitation. Brain slices were immersed in 2 µM ZnAF-2DA in Ringer solution containing 119 mM NaCl, 2.5 mM KCl, 1.3 mM MgSO_4_, 1.0 mM NaH_2_PO_4_, 2.5 mM CaCl_2_, 26.2 mM NaHCO_3_, and 11 mM D-glucose (pH 7.3) for 30 min, immersed in ice-cold choline-Ringer solution for 60 min, and transferred to a recording chamber filled with Ringer solution. The fluorescence of ZnAF-2 (Ex/Em: 488 nm/505–530 nm) was captured in the dentate gyrus with a confocal laser-scanning microscopic system.

### Propidium iodide (PI) staining

Fourteen days after ICV injection of Aβ_1-42_, the brain was quickly removed from the mice under anesthesia and immersed in ice-cold choline-Ringer. Coronal brain slices (400 µm) were prepared using a vibratome ZERO-1 (Dosaka Kyoto, Japan) in ice-cold choline-Ringer, which were continuously bubbled with 95% O_2_ and 5% CO_2_. The brain slices were bathed in PI in Ringer solution (7 µg/ml) for 30 min, bathed in Ringer solution for 30 min and transferred to a recording chamber filled with Ringer solution. PI fluorescence (Ex/Em: 535 nm/617 nm) was captured in the dentate gyrus with a confocal laser-scanning microscopic system.

### Fluoro-Jade B (FJB) staining

Fourteen days after ICV injection of Aβ_1-42_, the mice were anesthetized with chloral hydrate and perfused with ice-cold 4% paraformaldehyde in PBS, followed by removal of the brain and overnight fixation in 4% paraformaldehyde in PBS at 4 °C. Fixed brains were cryopreserved in 30% sucrose in PBS for 2 day and frozen in Tissue-Tek Optimal Cutting Temperature embedding medium. Coronal brain slices (30 µm) were prepared at -20 °C in a cryostat, picked up on slides, adhered at 50 °C for 60 min, and stored at -20 °C. The slides were first immersed in a solution containing 1% sodium hydroxide in 80% alcohol (20 ml of 5% NaOH added to 80 ml ethanol) for 5 min. This was followed by 2 min in 70% ethanol and 2 min in distilled water. The slides were then transferred to a solution of 0.06% potassium permanganate for 15 min on a shaker table to insure consistent background suppression between slices. The slides were then rinsed in distilled water for 2 min. The staining solution was prepared from a 0.01% stock solution of FJB that was made by adding 10 mg of the dye powder to 100 ml of distilled water. The stock solution and 0.1% 4',6-diamidino-2-phenylindole (DAPI) in distilled water were diluted with 0.1% acetic acid vehicle, resulting in a final dye concentration of 0.0004% FJB and 0.0001% DAPI in the staining solution. The staining solution was prepared within 10 min of use. The slides were bathed in the staining solution for 30 min and were rinsed for 2 min in each of three distilled water washes. Excess water was briefly removed by using a paper towel. The slides were placed at 50 °C for drying. The dry slides were twice immersed in xylene for 2 min before coverslipping with DPX, a non-aqueous, non-fluorescent plastic mounting media. FJB-positive cells in the unit area were measured in the dentate granule cell layer with a confocal laser-scanning microscopic system (Ex/Em: 480 nm/525 nm).

### In vivo long-term potentiation (LTP) recording

Male rats anesthetized with chloral hydrate (400 mg/kg) were placed in a stereotaxic apparatus. A bipolar stimulating electrode and a monopolar recording electrode made of tungsten wire attached to an injection cannula (internal diameter, 0.15 mm; outer diameter, 0.35 mm) were inserted to stimulate the perforant pathway of anesthetized rats and to record in the dentate granule cell layer, respectively, as reported previously [5, 8]. After stable baseline recording for at least 30 min, Aβ_1-42_ (25 µM) in saline was locally injected into the dentate granule cell layer of anesthetized rats at the rate of 0.25 μl/min for 4 min via an injection cannula attached to a recording electrode. LTP was induced by delivery of high-frequency stimulation (HFS; 10 trains of 20 pulses at 200 Hz separated by 1 s) 1 h after injection and recorded for 60 min.

### Object recognition memory

Rats were placed for 10 min into an open field, which was a 70 × 60 cm arena surrounded by 70 cm high walls, made of a black-colored plastic. Twenty-four hours after open field exploration, Aβ_1-42_ in saline was bilaterally injected via injection cannulas into the dentate granule cell layer of unanesthetized rats in the same manner as in vivo LTP recording section [5, 8]. One hour later, training was performed by placing each rat into the field, in which two identical objects were placed in two adjacent corners, 15 cm from the walls. Rats explored the objects for 5 min. One hour later, the rats explored the open field for 3 min in the presence of one familiar (A) and one novel (B) object. A recognition index calculated for each rat was expressed by the ratio T_B_/(T_A_ + T_B_) [T_A_ = time spent to explore the familiar object A; T_B_ = time spent to explore the novel object B].

### Data analysis

Differences between treatments were assessed by one-way ANOVA followed by post hoc testing using the Tukey’s test (the statistical software, GraphPad Prism 5). A value of *p* < 0.05 was considered significant. Data were expressed as means ± standard error. The results of statistical analysis are described in every figure legend.

## Results

### NYT-induced MT synthesis reduces Zn^2+^ level increased by Aβ_1-42_

MTs is a candidate, which reduces intracellular Zn^2+^ level increased by Aβ_1-42_, and newly synthesized MTs increase the capacity of capturing free Zn^2+^ [7, 8]. The present study was performed focused on the dentate granule cell layer because dentate gyrus neurons are the most vulnerable to Aβ_1-42_ toxicity in the hippocampus described below [7]. MT level was elevated in the dentate granule cell layer 2 days after administration of NYT diet (Fig. 1). Intracellular Zn^2+^ level, which was assessed by ZnAF-2 fluorescence, was elevated 1 h after ICV injection of Aβ_1-42_, while the increase was rescued by the pre-administration of NYT diet (Fig. 2). Because Aβ_1-42_ is taken up into hippocampal cells including dentate gyrus neurons [18], the increase in intracellular Zn^2+^ induced by Aβ_1-42_ is observed in the hippocampus, resulting in increase in ZnAF-2 intensity in the dentate gyrus by Aβ_1-42_ in the present study.

### Pre-intake of NYT diet rescues neuronal death

After ICV injection of Aβ_1-42_, neuronal death is preferentially observed in dentate gyrus neurons in the hippocampus [7] because of the high uptake of Aβ_1-42_ into dentate gyrus neurons [18]. We observed neuronal death in the dentate granule cell layer by using PI and FJB staining. PI fluorescence and FJB-positive cells were increased 14 days after ICV injection of Aβ_1-42_, while both increases were rescued by the pre-administration of NYT diet (Figs.3 and 4).

### Pre-intake of NYY diet rescues affected LTP and memory

**Fig. 5:**
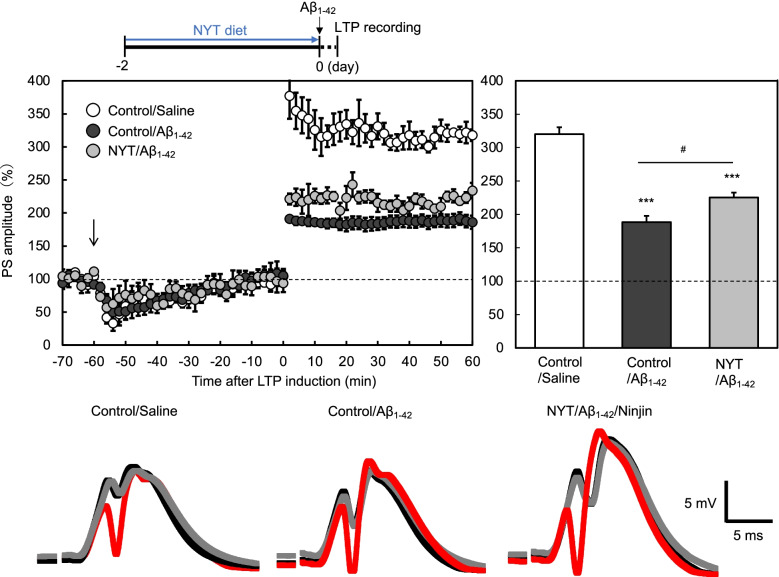
Attenuated LTP by Aβ_1-42_ is ameliorated by NYT diet. LTP recording was performed 2 days after administration of NYT diet to rats. LTP was induced 1 h after local injection of saline (vehicle) and Aβ_1-42_ in saline into the dentate granule cell layer via an injection cannula as shown by the arrow (upper-left). Averaged PS amplitudes for the last 10 min were represented as the magnitude of LTP (upper-right). ***, *p* < 0.001, vs. control/saline; ^#^, *p* < 0.05, vs. control/Aβ (Tukey’s test). Representative fEPSP recordings are shown at the time -70 min (before injection; black line), -20 min (after injection; grey line) and 60 min (after tetanic stimulation; red line) (lower). control/saline, *n* = 7 rats; control/Aβ, *n* = 7 rats; NYT/Aβ, *n* = 7 rats

In vivo LTP at the perforant pathway-dentate granule cell synapses was induced 1 h after local injection of Aβ_1-42_ into the dentate granule cell layer via an injection cannula attached to a recording electrode. LTP attenuated by Aβ_1-42_ was significantly ameliorated after oral administration of NYT diet for 2 days, while the amelioration was not complete (Fig. 5).

**Fig. 6:**
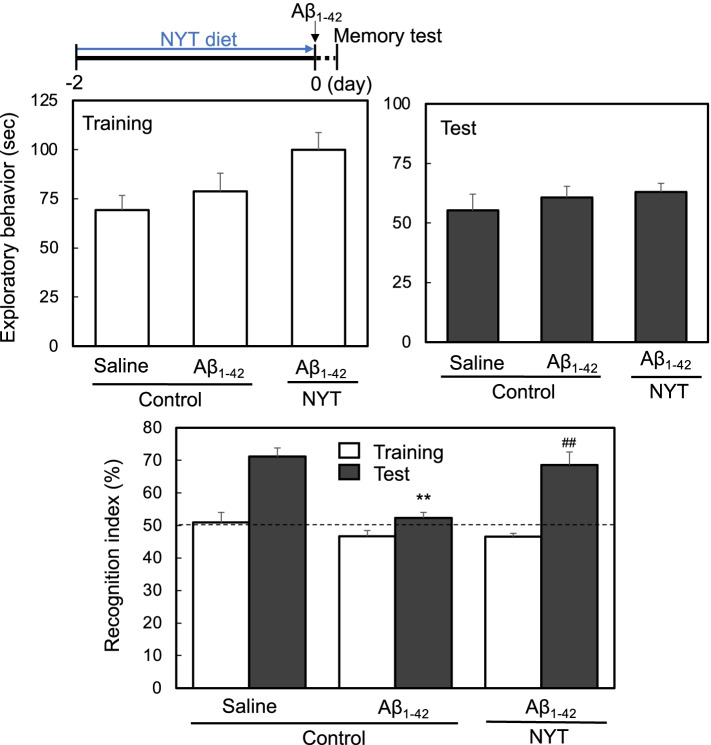
Exploratory behavior in the training and test of object recognition. The memory test was performed 1 h after local injection of saline (vehicle) and Aβ_1-42_ in saline into the dentate granule cell layer of rats via an injection cannula in the same manner as Fig. 5. The time of exploratory behavior in the field was measured in the training and test (middle). control/saline, *n* = 6 rats; control/Aβ, *n* = 7 rats; NYT/Aβ, *n* = 7 rats. One hour after training, the memory was evaluated as the recognition index (lower). **, *p* < 0.01, vs. control/saline in test; ^##^, *p* < 0.01, vs. control/Aβ in test (Tukey’s test). control/saline, *n* = 6 rats; control/Aβ, *n* = 7 rats; NYT/Aβ, *n* = 7 rats

In vivo performant pathway LTP is linked with object recognition memory [5]. When training of the object recognition test was done 1 h after local injection of Aβ_1-42_ into the dentate granule cell layer, the exploring time was not significantly affected by Aβ_1-42_ injection and NYT diet administration (Fig. 6). One hour later, the exploring time during the test was not also affected by Aβ_1-42_ injection and NYT diet administration (Fig. 6). In contrast, object recognition memory was impaired by Aβ_1-42_ injection, while the impairment was rescued by the intake of NYT diet (Fig. 6).

## Discussion

MTs capture 7 equivalents of Zn^2+^ and become a chemical form of Zn_7_MTs. The occupation of Zn^2+^-binding sites in MTs is correlated with Zn^2+^ concentration [19, 20]. Intracellular MTs are mainly a chemical form of Zn_5_MTs when intracellular Zn^2+^ is ~ 100 pM, an estimated basal concentration. In vivo K_d_ value of Zn^2+^ to Aβ_1-42_ is in the range of ~ 3–30 nM, while that to MTs is ~ 1 pM [6]. Thus, it is estimated that MTs can capture free Zn^2+^ derived from Aβ_1-42_ in the intracellular compartment. However, it was unclear whether the beneficial effect of NYT is directly linked with increased synthesis of MTs in the previous study [16]. When dexamethasone, an inducer of MT-I and MT-II, is intraperitoneally injected into rats once a day for 2 days, hippocampal MT level is significantly elevated 1 day after injection and reduces the increase in intracellular Zn^2+^ derived from Aβ_1-42_, followed by rescuing the affected LTP [8]. Aβ_1-42_-induced neurodegeneration is also rescued after the same treatment with dexamethasone [7]. On the basis of the data that the biological half-life of MTs is 18–20 h [17], it is estimated that the effective period of newly synthesized MTs on capturing toxic Zn^2+^ ferried by extracellular Aβ_1-42_ is a few days when MT synthesis is induced by the intake of NYT diet.

The present study indicates that pre-administration of NYT diet for 2 days increases synthesis of MTs and may reduce intracellular Zn^2+^ toxicity derived from Aβ_1-42_, resulting in protecting neuronal death by Aβ_1-42_. It is likely that increased synthesis of MTs plays a key role for the protecting effect of NYT. Aβ_1-42_-mediated neuronal death is rescued after co-injection of extracellular (CaEDTA) and intracellular (ZnAF-2DA) Zn^2+^ chelators [7], supporting that Zn^2+^ release from intracellular Zn-Aβ_1-42_ complexes plays a key role for neuronal death.

Hippocampus-related memory of object recognition is affected when in vivo perforant pathway LTP is attenuated after local injection of Aβ_1-42_ into the dentate granule cell layer [5, 7, 8]. In the present study, in vivo perforant pathway LTP, which was successfully recorded after local injection of Aβ_1-42_ into the dentate granule cell layer as reported previously [5, 7, 8], was impaired by Aβ_1-42_, while the impairment was ameliorated by pre-administration of NYT diet for 2 days. Furthermore, the pre-administration of NYT diet for 2 days rescued object recognition memory loss by Aβ_1-42_, suggesting that increased synthesis of MTs plays a key role for the rescuing effect of NYT on memory loss by Aβ_1-42._ Aβ_1-42_-mediated impairments of LTP and memory are also rescued after co-injection of extracellular (CaEDTA) and intracellular (ZnAF-2DA) Zn^2+^ chelators [5], supporting that Zn^2+^ release from intracellular Zn-Aβ_1-42_ complexes plays a key role for hippocampal dysfunction.

There is no evidence on MT synthesis in the brain by Kampo medicines. NYT is traditionally used for the patients with insomnia, neurosis, and anorexia [21], suggesting that NYT components may pass through the blood–brain barrier and increase synthesis of MTs in the brain. Unfortunately, there is no evidence on inducers to facilitate MT synthesis in the brain, which are secure for the brain function. The reason is that most MT inducers are not taken up into the brain parenchyma cells because of impermeability against the blood–brain barrier [22]. Exogenous catecholamines including isoproterenol, which cannot pass through the blood–brain barrier, induces MTs in peripheral tissues, e.g., the liver and kidney [23–25]. Isoproterenol, an adrenergic β receptor agonist, enhances MT synthesis in the dentate gyrus and cancels neurodegeneration via intracellular Zn^2+^ toxicity after ICV co-injection of Aβ_1-42_ and isoproterenol [26]. It is estimated that MT synthesis is enhanced by adrenergic β receptor-mediated signaling after the intake of NYT diet and contributes to ameliorating Aβ_1-42_ toxicity in the brain. It is necessary to clarify NYT components to lead to adrenergic β receptor-mediated signaling.

## Conclusion

The present study suggests that MT synthesis by NYT contributes to protecting neuronal death in the dentate gyrus and memory loss after exposure to Aβ_1-42_. It is likely that MT synthesis by NYT components protectively act on hippocampal function.

## Data Availability

All data supporting the conclusions are included in the manuscript.

## References

[1] Scheff SW, Price DA, Schmitt FA, Mufson EJ (2006). Hippocampal synaptic loss in early Alzheimer’s disease and mild cognitive impairment. Neurobiol Aging.

[2] Crews L, Masliah E (2010). Molecular mechanisms of neurodegeneration in Alzheimer’s disease. Hum Mol Genet.

[3] Go´mez-IslaPrice TJL, McKeel DW, Morris JC, Growdon JH, Hyman BT (1996). Profound loss of layer II entorhinal cortex neurons occurs in very mild Alzheimer’s disease. J Neurosci.

[4] Brouillette J, Caillierez R, Zommer N, Alves-Pires C, Benilova I, Blum D, De Strooper B, Buée L (2012). Neurotoxicity and memory deficits induced by soluble low-molecular-weight amyloid-β1-42 oligomers are revealed in vivo by using a novel animal model. J Neurosci.

[5] Takeda A, Nakamura M, Fujii H, Uematsu C, Minamino T, Adlard PA, Bush AI, Tamano H (2014). Amyloid β-mediated Zn^2+^ influx into dentate granule cells transiently induces a short-term cognitive deficit. PLoS ONE.

[6] Takeda A, Tamano H, Tempaku M, Sasaki M, Uematsu C, Sato S, Kanazawa H, Datki ZL, Adlard PA, Bush AI (2017). Extracellular Zn^2+^ is essential for amyloid β_1-42_-induced cognitive decline in the normal brain and its rescue. J Neurosci.

[7] Tamano H, Takiguchi M, Tanaka Y, Murakami T, Adlard PA, Bush AI, Takeda A (2020). Preferential neurodegeneration in the dentate gyrus by amyloid β_1-42_-induced intracellular Zn^2+^ dysregulation and its defense strategy. Mol Neurobiol.

[8] Takeda A, Tamano H, Hashimoto W, Kobuchi S, Suzuki H, Murakami T, Tempaku M, Koike Y, Adlard PA, Bush AI (2018). Novel defense by metallothionein induction against cognitive decline: from amyloid β_1-42_-induced excess Zn^2+^ to functional Zn^2+^ deficiency. Mol Neurobiol.

[9] Tamano H, Takiguchi M, Saeki N, Katahira M, Shioya A, Tanaka Y, Egawa M, Fukuda T, Ikeda H, Takeda A (2021). Dehydroeffusol prevents amyloid β_1-42_-mediated hippocampal neurodegeneration via reducing intracellular Zn^2+^ toxicity. Mol Neurobiol.

[10] Davies P, Maloney AJ (1976). Selective loss of central cholinergic neurons in Alzheimer’s disease. Lancet.

[11] Bartus RT, Dean RL, Beer B, Lippa AS (1982). The cholinergic hypothesis of geriatric memory dysfunction. Science.

[12] Whitehouse PJ, Price DL, Struble RG, Clark AW, Coyle JT, Delon MR (1982). Alzheimer’s disease and senile dementia: loss of neurons in the basal forebrain. Science.

[13] Summers WK, Majovski LV, Marsh GM, Tachiki K, Kling A (1986). Oral tetrahydroaminoacridine in long-term treatment of senile dementia. Alzheimer type N Engl J Med.

[14] Rogers SL, Farlow MR, Doody RS, Mohs R, Friedhoff LT (1988). Donepezil Study Group. A 24-week, double-blind, placebo-controlled trial of donepezil in patients with Alzheimer’s disease. Neurology.

[15] Kudoh C, Arita R, Honda M, Kishi T, Komatsu Y, Asou H, Mimura M (2016). Effect of ninjin'yoeito, a Kampo (traditional Japanese) medicine, on cognitive impairment and depression in patients with Alzheimer's disease: 2 years of observation. Psychogeriatrics.

[16] Tamano H, Tokoro H, Murakami D, Furuhata R, Nakajima S, Saeki N, Katahira M, Shioya A, Tanaka Y, Egawa M, Takeda A (2021). Preventive effect of Ninjin-yoei-to, a Kampo medicine, on amyloid β_1-42_-induced neurodegeneration via intracellular Zn^2+^ toxicity in the dentate gyrus. Exp Anim.

[17] Cousins RJ (1979). Metallothionein synthesis and degradation: relationship to cadmium metabolism. Environ Health Perspect.

[18] Tamano H, Oneta N, Shioya A, Adlard PA, Bush AI, Takeda A (2019). In vivo synaptic activity-independent co-uptakes of amyloid β_1-42_ and Zn^2+^ into dentate granule cells in the normal brain. Sci Rep.

[19] Krężel A, Maret W (2007). Dual nanomolar and picomolar Zn(II) binding properties of metallothionein. J Am Chem Soc.

[20] Krężel A, Maret W (2017). The Functions of Metamorphic Metallothioneins in Zinc and Copper Metabolism. Int J Mol Sci.

[21] Goswami C, Dezaki K, Wang L, Inui A, Seino Y, Yada T (2019). Ninjin-yoeito activates ghrelin-responsive and unresponsive NPY neurons in the arcuate nucleus and counteracts cisplatin-induced anorexia. Neuropeptides.

[22] Ebadi M, Iversen PL, Hao R, Cerutis DR, Rojas P, Happe HK, Murrin LC, Pfeiffer RF (1995). Expression and regulation of brain metallothionein. Neurochem Int.

[23] Brady FO, Helvig B (1984). Effect of epinephrine and norepinephrine on zinc thionein levels and induction in rat liver. Am J Physiol.

[24] Beattie JH, Wood AM, Trayhurn P, Jasani B, Vincent A, McCormack G, West AK (2000). Metallothionein is expressed in adipocytes of brown fat and is induced by catecholamines and zinc. Am J Physiol Regul Integr Comp Physiol.

[25] Bobillier-Chaumont S, Maupoil V, Berthelot A (2006). Metallothionein induction in the liver, kidney, heart and aorta of cadmium and isoproterenol treated rats. J Appl Toxicol.

[26] Kawano Y, Tamura K, Egawa M, Tamano H, Takeda A (2022). Isoproterenol, an adrenergic β receptor agonist, induces metallothionein synthesis followed by canceling amyloid β_1-42_-induced neurodegeneration. Biometals.

